# The Effect of Solidification Rate on the Corrosion Resistance of Die-Cast AZ91 Magnesium Alloy

**DOI:** 10.3390/ma15031259

**Published:** 2022-02-08

**Authors:** Kwangmin Choi, Jaehyuck Shin, Heon Kang

**Affiliations:** 1 Department of Materials Science and Engineering, Yonsei University, 50 Yonsei-ro Seodaemungu, Seoul 03722, Korea; ckm88wkd@yonsei.ac.kr; 2 Materials Technology R&D Division, Korea Automotive Technology Institute (KATECH), 303 Pungse-ro Pungse-myeon, Cheonan-si 31214, Korea; jhshin@katech.re.kr; 3 School of Advanced Materials Engineering, Kookmin University, 77 Jeongneung-ro Seongbuk-gu, Seoul 02707, Korea

**Keywords:** magnesium, solidification rate, corrosion resistance, AZ91, die casting

## Abstract

To increase the utilization of die-cast Mg alloys with various shapes in a variety of environments, the corrosion behaviors of commercial die-cast Mg alloys with different thicknesses were investigated in neutral and alkali solutions at ambient temperature. A decrease in the thickness of a specimen leads to an increase in cooling and solidification rates, which, in turn, decreases the size of the eutectic β phases and the interphase distance, thus improving the hardness of the specimen. Specimens with relatively large β phases were more corroded under neutral conditions due to severe galvanic corrosion at the interface between α-Mg and the β phases, whereas they were protected by passivation films formed on the substrate in the alkaline solution. However, in the case of the alloy with thin thickness and high solidification rate, the fine β phases improved corrosion resistance by forming a net structure that acted as a barrier to corrosion propagation of the α matrix. These results suggest that the size and distribution of the eutectic phases should be appropriately controlled, depending on the environment.

## 1. Introduction

The energy efficiency of designs can be enhanced by using high-performance lightweight structural materials. Magnesium (Mg), which is of interest due to its low specific gravity (1.74 g/cm^3^), holds great potential for use in automotive and aerospace applications [1,2]. Die-casting techniques are frequently used in the manufacture of Mg components because they can produce high-quality parts at a high production rate and reasonable cost [3]. Commercial AZ91 (Mg-9 wt.% Al-1 wt.% Zn) alloy is the most widely used Mg-Al die-cast alloy, due to its high fatigue strength and excellent castability for even the most complex and thin-walled parts [4].

Unfortunately, the low corrosion resistance of Mg and its alloys limits its widespread use in many applications, particularly in those involving atmospheres that contribute to pitting corrosion [5,6]. Generally, the corrosion reaction of Mg and its alloys are similar in both neutral and alkaline media, and the overall reaction can be expressed as

Mg + 2H_2_O → Mg(OH)_2_ + H_2_(g) (1)

However, this reaction formula is only a general description and does not consider the negative differential effect, a common phenomenon in the anodic dissolution of Mg alloys. The corrosion properties of Mg alloys are closely related to the chemical compositions and activities of their impurities [7]. Therefore, the corrosion resistance of Mg and its alloys can be improved by eliminating any heavy metal impurities, such as iron, nickel, and copper, during the cleaning process [8]. In the Mg-Al-based alloy system, the β phase acts as a cathode and exhibits good passive behavior in a broad pH range. Following the dissolution of the anodic α-Mg, the β phase may inhibit corrosion by acting as a barrier layer. According to previous studies [9,10], the role of the β phase in the corrosion process is related to its composition, size, and distribution. For example, die-cast AZ91 has been reported to show higher corrosion resistance and better passivation than ingot AZ91, because the fine grain structure and β phases of the die-cast alloy make it more cathodic than the ingot alloy [11]. The AZ91 alloy contains a large fraction of the intermetallic compounds of the β-Mg_17_Al_12_ phase, usually distributed along the grain boundary of the α-Mg phase (matrix). Hence, fine β phases with sizes of only tens of micrometers can play significant roles in die-cast AZ91 [12,13], and the size and distribution of the β phases in die-cast alloys are greatly affected by the cooling rate.

In recent years, many electronic devices are being applied to improve the convenience of transport such as automotive. Due to the application of a large number of electronic devices in transport, a reduction in the weight of the devices is required, and the weight reduction leads to the reduction in the thickness of the heat exchangers. One of the solutions for this purpose is a die-casting method that can meet the required mechanical properties with a thinner thickness. However, studies on the effect of the cooling rates, the morphology of the β phases, and the properties of die-cast AZ91 in a corrosive environment have not been sufficiently studied.

In the present study, AZ91 alloy specimens with various thicknesses were fabricated by high-pressure die casting (HPDC), and the influence of the size and distribution of β phases on the corrosion resistance of the alloy was investigated in aqueous solutions containing chloride and hydroxide ions. The relationships between the microstructures and corrosion behaviors of the specimens were determined by examining their microstructures before and after corrosion.

## 2. Materials and Methods

A commercial AZ91 alloy (Mg-9 wt. % Al-1 wt. % Zn, Magnesium Elektron, Manchester, UK) was used as the starting material in this study. The alloy was melted at 700 °C in a boron nitride (BN)-coated low carbon steel crucible set in an electrical furnace in a dynamic SF_6_+ CO_2_ (SF_6_:CO_2_ = 1:10) atmosphere. After holding at this temperature for 30 min, the melt temperature was lowered to the target casting temperature (670 °C) for HPDC. The melt was pressurized by a plunger (φ 55 mm) and moved to a cold chamber preheated at 200 °C. The plunger speed and pressure were 2 m/s and 40 MPa, respectively. Four types of specimens with various thicknesses (i.e., 2.5, 5, 10, and 20 mm) were produced, and these are listed in Table 1.

A back-scattered electron (BSE) detector set in a scanning electron microscope (SEM, JEOL, JSM-7001, Akishima, Japan) was used to examine the microstructures of the specimens before and after the corrosion tests. The volume and distribution of the eutectic phases were analyzed using an image analyzer (Image-Pro Plus, Media Cybernetics Inc., Rockville, MD, USA) The specimens were prepared for microstructure observation by mechanical polishing with SiC paper (up to 2000 grit). To verify the relationships between the microstructure and the mechanical properties, the hardness values of all of the specimens were measured at various positions in the thickness direction using the Vickers hardness (Hv) test with an indenter load of 300 gf.

The corrosion potential and current density were measured using a potentiostat/galvanostat (AMETEK, Versa STAT 3, Pittsburgh, PA, USA). Before testing, the die-cast specimens were polished with SiC paper (up to 2000 grit), degreased with acetone, and washed with distilled water. The tests were performed in 0.1 M NaCl and 0.1 M NaOH solutions at a temperature of 25 ± 1 °C using a conventional three-electrode cell consisting of a carbon plate as a counter electrode and a Calomel reference electrode (AMETEK, Pittsburgh, PA, USA), giving a circular testing area of 28.26 m^2^. The open-circuit potentials of the specimens were analyzed for 3000 s. The potential ranges of the corrosion tests in the 0.1 M NaCl (pH 7.0) and 0.1 M NaOH (pH 12.4) solutions were between −2.0 and −1.0 V, and −1.4 and 0 V, respectively. The scanning rate was 0.1 mV/s for all tests. The surface products were analyzed by X-ray diffraction (XRD, Rigaku, CN2301, Akishima, Japan) using Cu Kα radiation (λ = 1.5416 Å) filtered through Ni foil. The XRD patterns were collected over 20–80° with a scan speed of 0.02°/s.

## 3. Results

**Figure 1:**
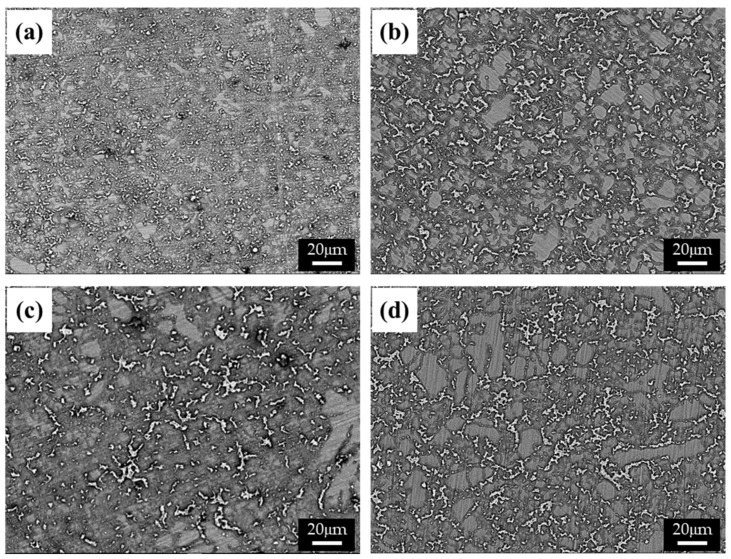
BSE images of the AZ91 alloys with various thicknesses: (**a**) 2.5, (**b**) 4, (**c**) 6, and (**d**) 10 mm.

The cooling rate of die-cast specimens, which generally varies with the thickness of the specimen, affects the size and distribution of the β phase. The microstructures of specimens with various thicknesses (i.e., 2.5 (Figure 1a), 5 (Figure 1b), 10 (Figure 1c), and 20 mm (Figure 1d)) were observed using a BSE detector in an SEM, as shown in Figure 1. The structure of the β phase in a specimen becomes greatly refined as the thickness of the specimen decreases, due to the increased cooling rate and resulting in a higher solidification rate. This relation can be described as follows [14]:

(2) $$v={\left(d_{2}/k\right)}^{-3}$$

where *v* is the solidification rate, and *d*_2_ and *k* are the secondary dendrite arm spacing and the constant, respectively. Rapid solidification in a thin specimen may lead to under-cooling of the melt, which increases the number of effective nuclei while decreasing their growth rate. As a result, both grains and dendrite structures are refined.

The cooling rate (*T_c_*) during die casting can be calculated as follows [15]:

(3) $$T_{c}\;=\;\left(T_{i}-T_{f}\right)/\left(t_{f}\right)$$

where *T_i_* and *T_f_* are the initial and final temperatures, respectively, and *t_f_* is the solidification time. Using Thermo-calc software, the melting (*T_i_*) and solidification (*T_f_*) temperatures of the AZ91 alloy were calculated to be 580 and 470 °C, respectively. The solidification time of each sample was experimentally measured to be 1.5, 3.2, 4.8, and 9.4 s for specimens 1, 2, 3, and 4, respectively. According to Equation 3, the cooling rate in specimens 1, 2, 3, and 4 was estimated to be 73.3, 34.3, 22.9, and 11.7 °C/s, respectively; the cooling rate of specimen 1 was approximately 7 times faster than that of specimen 4, resulting in a large difference in the size and distribution of the β phases among the specimens.

The distance between the β phases in the BSE images was estimated using an image analyzer, as summarized in Table 1. The distance between the phases was five times shorter in specimen 1 than in specimen 4, possibly due to the higher solidification rate [16]. The volume fraction of the α matrix also increased as the solidification rate increased, as shown in Table 1. The alloying elements can be supersaturated in the α-Mg at a high solidification rate, limiting the formation of eutectic phases.

**Figure 2:**
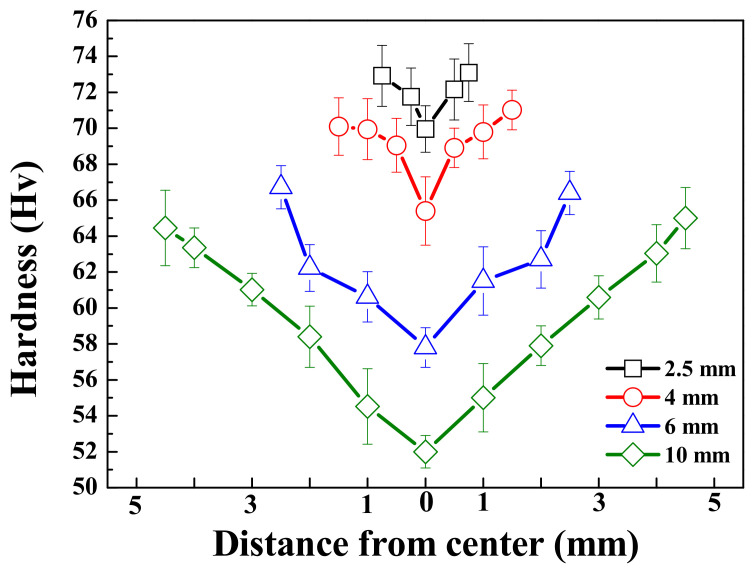
Vickers’ hardness values as a function of distance from the center of the AZ91 alloy with various thicknesses. Each lines with black, red, blue, and green indicate the thickness of samples 2.5, 4, 6, and 10 mm, respectively.

The Vickers hardness of all specimens, measured in the thickness direction, is shown in Figure 2. For all samples, the hardness was measured to be highest at the surface and found to decrease as the distance from the surface increased. Due to the rapid solidification rate at the surface relative to the center, the microstructure and hardness may be finer and higher, respectively, near the surface. In contrast, despite having a lower volume fraction of β phases, thinner specimens exhibit higher hardness, both near the surface and at the center, as the interphase spacing has a more dominant effect on the hardness than does the volume of the β phases. This can be described by the Orowan equation as [17]

(4) $$H_{v}\;=\;\left(0.13G_{m}b/d\right)lnr/b$$

where d is the distance between the phases, $G_{m}$ is the shear modulus of the matrix, b is the Burgers vector, and r is the dispersed radius of the β-phase. For a given alloy, the shear modulus of the matrix and the Burgers vector can be considered constant. Hence, when the microstructures are refined, the distance between the phases and the size of each phase becomes smaller, leading to an increase in hardness. This observation is comparable with previous reports of die-cast AZ91 alloys generally exhibiting higher hardness (70–80 Hv) than AZ91 alloys manufactured by sand casting or squeeze casting (~60 Hv). This phenomenon is due to the formation of fine β phases throughout the specimen resulting from the rapid cooling rate [16].

**Figure 3:**
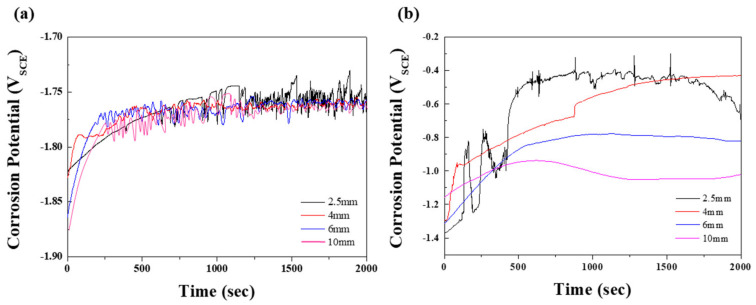
Open-circuit potentials, varied according to test time, of the specimens with various thicknesses in (**a**) 0.1 M NaCl and (**b**) 0.1 M NaOH solution.

To study the corrosion properties of the specimens, electrochemical tests were performed in 0.1 M NaCl and 0.1 M NaOH solutions. Figure 3 shows the open-circuit potentials (E_OCP_) at various times; the values are summarized in Table 2. Generally, the E_OCP_ values of magnesium alloys in neutral solutions initially rose sharply, followed by fluctuation. This behavior indicates that the surface of the film is attacked by chloride ions before becoming covered by a protective film from the corrosion product [18]. In Figure 3a, the E_OCP_ values of specimens 2, 3, and 4 are shown to stabilize after rising slightly. In contrast, the potential values of specimen 1 showed a relatively slow incremental increase until 700 s, followed by a dramatic fluctuation due to galvanic corrosion induced by the numerous β phases, that is, the protective film was repeatedly broken and recovered. Although specimens 2, 3, and 4 exhibited similar tendencies to specimen 1, the time for the slow-down of the potential fluctuation for specimen 1 was longer. The effect of the distribution of eutectic phases on the corrosion resistance was demonstrated in an alkaline solution, in which a more protective film was formed by the reaction: Mg^+^ + 2(OH) → Mg(OH)_2_ + e^−^ [19]. As shown in Figure 3b, the E_OCP_ values of all specimens in the 0.1 M NaOH solution gradually increased, with saturation occurring after 1500 s, followed by a drop as the distance between the β phases increased. For specimen 1, which had the greatest volume fraction of β phases among all specimens, early corrosion was delayed, giving a relatively slow incremental increase in E_OCP_. However, once corrosion started to occur, the potential fluctuated significantly due to the extensive galvanic corrosion between the β phases and the α matrix, as observed in the neutral solution. During corrosion of specimen 1, continuous breaking of the protective film may occur, due to galvanic corrosion causing the Mg to dissolve faster in this film than in the passive film. Therefore, specimen 1, which had the largest volume fraction of the exposed β phase, showed the highest potential in both solutions. In nature, Mg(OH)_2_ is nonuniformly formed on the surface of magnesium, resulting in poor corrosion resistance. In NaOH solution, Mg(OH)_2_ passivation film on the specimen was formed when OH^−^ was released from NaOH. Since the size of Mg^2+^ is smaller than that of Na^+^, the Mg–OH bond is stronger than the Na–OH bond. As can be inferred from Figure 3b, corrosion potential was low due to insufficient provision of OH^−^ ions in the early stage of the OCP test in NaOH condition. As the supply of OH^−^ ions increase over time, the potential gradually increases due to densely formed Mg(OH)_2_ film.

**Figure 4:**
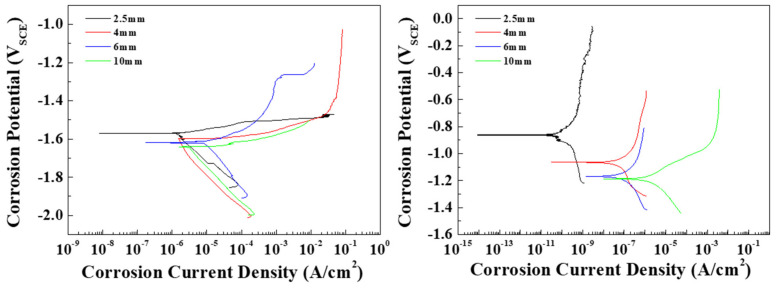
Potentiodynamic polarization curves of the specimens with various thicknesses in (**a**) 0.1 M NaCl and (**b**) 0.1 M NaOH solutions.

To observe the effects of the distribution of the β phase on the corrosion resistance of the AZ91 alloy in conflicting conditions, tests were conducted to determine the corrosion potential and current density of the specimens in 0.1 M NaCl and 0.1 M NaOH solutions. The Pourbaix potential-pH diagram of magnesium shows that possible protection occurs at a high pH (from 8.5), where the activity of magnesium ions is equal to 1 mol at 25 °C, and magnesium is dissolved over a wide pH range (under 8.5) [20]. In this study, since the pH values of 0.1 M NaCl and 0.1 M NaOH are 7 and 12.4, respectively, the die-cast specimens can be expected to be either corroded or protected in these solutions. Tafel curves for each specimen are shown in Figure 4, and the measured corrosion potentials (E_corr_) and corrosion current densities (I_corr_) are summarized in Table 2. Figure 4a shows the cathodic and anodic polarization curves of the specimens in the 0.1 M NaCl solution. The pitting potential was determined from the curves as the potential at which the current density reached 100 μA/cm^2^. All specimens showed a similar cathodic polarization behavior, in which the cathodic current density rose rapidly to around 1 μA/cm^2^. In contrast, the anodic polarization curves were found to depend on the microstructure of the specimen. Although the standard potential (E_0_) values in all specimens were around 1.60 V, the E_corr_ values showed a slight increase as the solidification rate increased. Since the potentials of the α-Mg (around −1.73 V) and the β phases (around −1.28 V) contribute to E_corr_, the E_corr_ value increases with the β-phase volume fraction. The pitting potentials of all specimens were located between approximately −1.5 and −1.6 V, that is, passivation films were not apparent in the specimens, and the anodic current densities increased with the applied potentials. In AZ91 alloys, the potential difference between the α-Mg and the β phase induces galvanic corrosion. Thin specimens with numerous β phases show a higher I_corr_ than thick specimens. Figure 4b shows the curves of the specimens in 0.1 M NaOH. The cathodic polarization behavior was similar in all specimens in that the cathodic current density rose rapidly and then slowly increased as the E_corr_ value was approached. In general, the specimens in the alkaline solution are spontaneously passivated by Mg(OH)_2_, and the chemical dissolution of Mg(OH)_2_ occurs when the dissolved OH^−^ ions are absorbed on the corroded areas [21]. Moreover, the corrosion potentials of the specimens in the alkaline solution were higher than those of the specimens in the neutral solution due to the restriction of the galvanic corrosion by a strong passivation film. In specimens 1, 2, and 3, no pits were observed on the surface when the passive current density was lower than 1 μA/cm^2^. Pits appeared in specimen 4 at a current density of around 80 μA/cm^2^. The noble shift in E_corr_ was attributed to the volume fraction of the β phase. Therefore, specimen 1, with fine β phases, had the highest E_corr_ and the lowest I_corr_ of all the specimens. In general, ingot AZ91 exhibits an E_corr_ of ~−1.5 V and an I_corr_ of ~1212 μA/cm^2^ in neutral solutions. However, fine β phases are continuously distributed at the boundaries of small grains in die-cast AZ91 due to rapid cooling rates, and these can effectively act as barriers to inhibit the corrosion of α-Mg [11]. As a result, specimen 1 showed a significantly lower I_corr_ in comparison with general ingot AZ91 alloys. The corrosion rate as the penetration rate can be obtained using the corrosion current density. The conversion equations of the rate are as follows [22]:

(5) $$mm/yr\;:0.00327\times (\left(EW\times i_{corr})/D\right)$$

where *mm/yr*, *EW*, *i_corr_*, and *D* are the mils penetration per year, the metric equivalent millimeter per year, equivalent weight, corrosion current density, and density of the alloy, respectively. As EW [23] and density of the AZ91 alloy are 11.89 g/eq and 1.81 g/cm^3^, respectively, the penetration rate(mm/yr) of all specimens in 0.1 M NaCl and 0.1 M NaOH were calculated and are shown in Table 3. The penetration rate in 0.1 M NaCl increases rapidly from specimen 3, with a thickness of 6 mm. Specimen 4 has a penetration rate that is almost four times faster than that of specimen 1 in the 0.1 M NaCl condition. In the 0.1 M NaOH condition, the phenomenon occurs prominently in specimen 1, with a thickness of 2.5 mm, while the penetration rate rapidly increases at a thickness of 4 mm or more.

**Figure 5:**
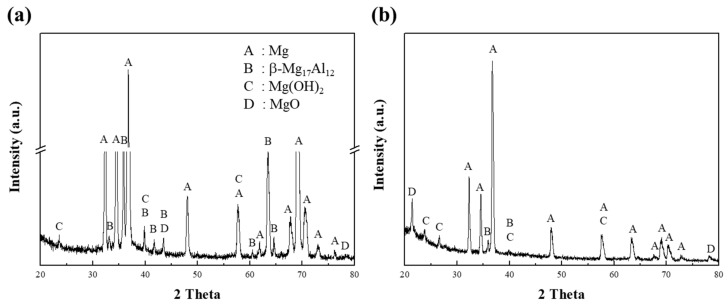
The XRD patterns, obtained from the corroded surface of specimen 1 after corrosion tests in (**a**) 0.1 M NaCl and (**b**) 0.1 M NaOH solutions.

After exposure of specimen 1 to the corrosion media, the corroded regions at the surface of the specimen were analyzed by XRD, as shown in Figure 5. In Figure 5a, the XRD pattern of specimen 1 corroded in a 0.1 M NaCl solution shows clear peaks corresponding to the α-Mg and β phases. The peaks for the β phases were easily detected due to greater corrosion at the interface between the α matrix and the β phases. In the initial stage of the corrosion of AZ91 alloys, the β phase can play a dual role in the dissolution behavior [24], in that it can act as either a galvanic cathode or as a kinetic barrier to dissolution [11]. Lunder et al. [25] reported that the corrosion potential of the β phase in 5% NaCl saturated with Mg(OH)_2_ is cathodic and occurs at about 490 and 420 mV for pure Mg and AZ91 alloys, respectively. Since this phase is highly cathodic for α-Mg, hydrogen evolution takes place preferentially at the β phase, thus making it an effective cathode. In contrast, the present study showed that the intensities of the β-phase peaks were no higher in the alkaline solution than in the neutral solution, as shown in Figure 5b for Mg(OH)_2_ and MgO. The corrosion behavior of pure Mg in the alkaline solution is such that strong, homogeneous films are formed by the OH^−^ ions supplied by the solution, whereas in the neutral solution, the Mg^2+^ ions are mainly responsible for the corrosion.

**Figure 6:**
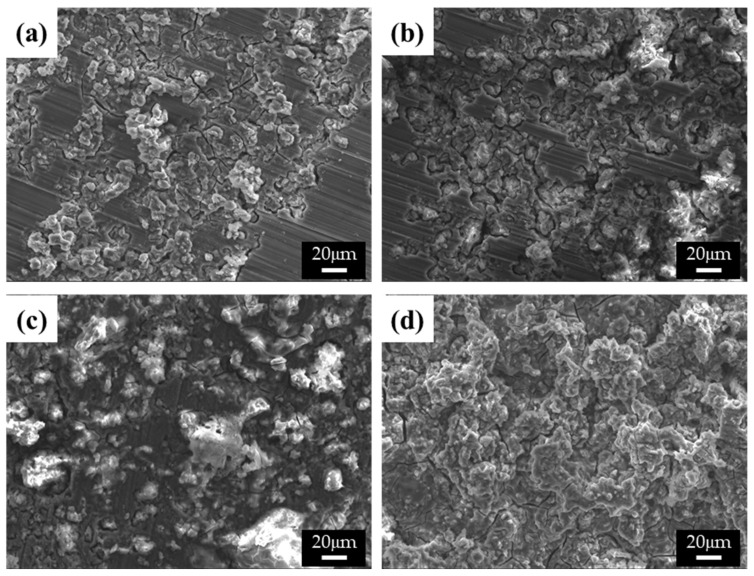
SEM images of the corroded surface of the specimens after corrosion tests in 0.1 M NaCl solutions: (**a**) specimen 1, (**b**) specimen 2, (**c**) specimen 3, and (**d**) specimen 4.

**Figure 7:**
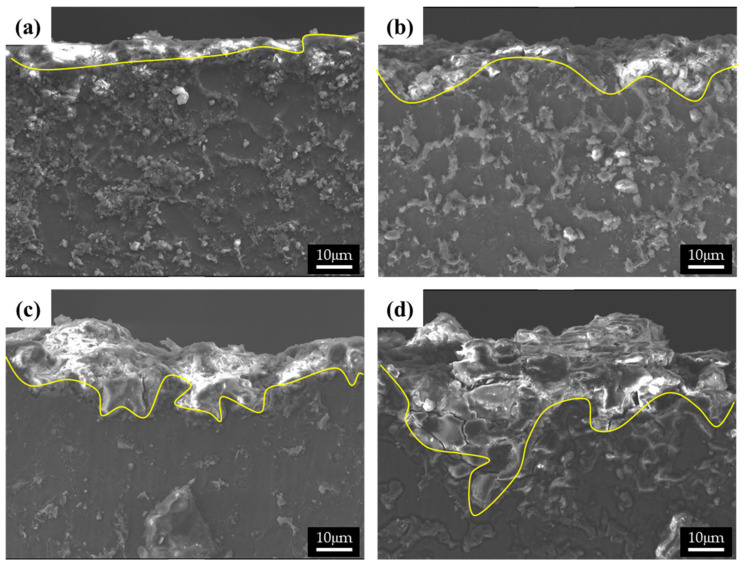
SEM images of the cross-section of the corroded specimens after corrosion tests in 0.1 M NaCl solutions: (**a**) specimen 1, (**b**) specimen 2, (**c**) specimen 3, and (**d**) specimen 4.

The differences in the corrosion behavior of specimens with varying β-phase spacing were uncovered by observing the corroded surfaces of the specimens in both 0.1 M NaCl and 0.1 M NaOH solutions, as shown in Figure 6 and Figure 7, respectively. The corrosion process is greatly affected by the solution ions when the surfaces of the alloys are covered with thin protective films [17]. In Figure 6a,b, microstructures with finer β phases show ring-like cracks (RLCs) for specimens 1 and 2. The diameters of the RLCs in specimen 1 are less than 10 μm; cracks are formed around the β phases because of the high chemical potential energy at the interface between the β phases and α matrix, thereby giving the RLCs the same spatial distribution as the β phases. Nearly all the single RLCs evolved into larger cracked and eroded areas. In specimen 2, larger RLCs (≤20 μm) formed at the phase agglomeration, and some pits were observed. The diameter of the pits was related to the conglomerates. The spatial distribution of the RLCs also indicated that corrosion occurred at the interface between the α-Mg and β phases, and the corrosion rates and the number of corrosion products increased with the distance between the phases. Some RLCs more than 50 μm in size could be observed in specimen 3. Deep corrosion cracks and corrosion products, such as magnesium oxides, were also observed. RLCs could not be observed in specimen 4, as these were covered with large corrosion products. In galvanic corrosion, the magnesium ions dissolved from the α matrix easily form corrosion products, and the number of ions increases with a larger matrix size, that is, the α matrix is corroded more quickly than the β phase is inhibited. As evident from the results of the curve shown in Figure 4a, specimen 3 was found to have improved corrosion properties above the corrosion potential, as numerous and large corrosion products were found to cover its surface, which was found to be beneficial for protection against corrosion. In these corrosion tests, since the surface of the specimen was mainly corroded and most of the pores were generated in the center of the casting alloy, the corrosive effect by pores is negligible, though the cooling rate is changed.

The cross-sectional images of the corroded specimens following the corrosion tests in the neutral solution are shown in Figure 7. The appearance of corroded surfaces in the specimens with fine β phases is different from that of the specimens with coarse phases. The cross-sectional image of specimen 1 in Figure 7a shows that the corroded layer is uniform, approximately 5 μm thick, and without pits. The image of specimen 2 in Figure 7b also shows a relatively uniform corroded layer with a few pits (approximately 10 μm in depth). The specimens with large phases show complex corrosion morphologies. In contrast, specimen 3 shows a deeply dug surface (Figure 7c). The formation of the morphology of the specimen can be described in the following steps: (1) Deep pits develop in which many cracks are formed, (2) corrosion progresses around the pits and cracks, and (3) the corroded regions are eliminated. The corrosion products in specimen 4 possessed large phases that were still present on the surface, as the α-Mg matrix of specimen 4 was larger than that of specimen 3. Thus, deep pits and numerous cracks were observed in specimen 4. A shallow pit penetrated about 18 μm into the matrix, which is close to the distance between the β phases. Furthermore, deeply and locally formed corrosion regions led to the formation of crevices approximately 50 μm in size, especially when the β-phase intervals were larger.

The β phase causes different phenomena depending on the microstructure in corrosion. In general, the β phase can act as a galvanic cathode because the cathodic hydrogen evolution on the β-phase surface is much stronger than that on the surface of the α phase. However, when the grains are very fine, the volume fraction of the β phase is high enough, and the β phase is nearly continuous similar to a net over the matrix, then the β-phase net shown in Figure 1a,b acts mainly as an anodic barrier against the corrosion attack and delays the acceleration of corrosion [11]. Therefore, the alloy with a net-phase structure (interphase distance: less than 10 μm) denotes superior corrosion resistance, almost three times better than that of above 10 μm in NaCl condition, as shown in Table 2.

**Figure 8:**
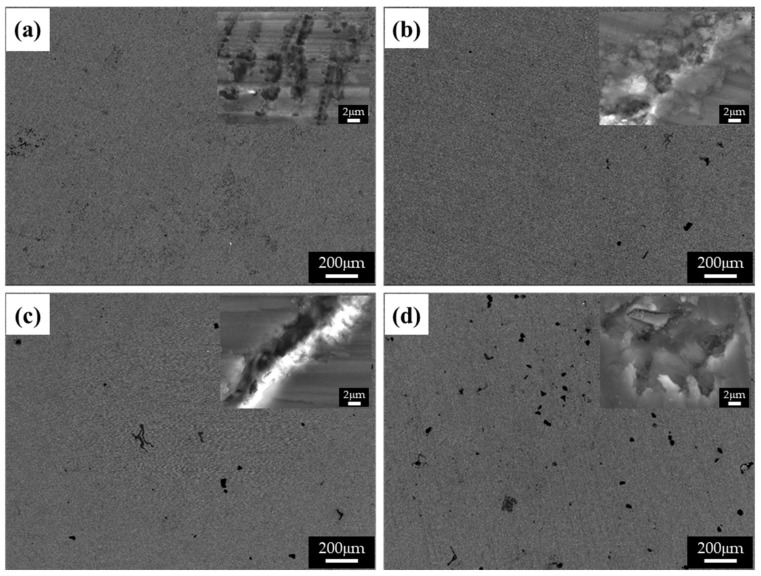
BSE images of the corroded surface of the specimens after corrosion tests in 0.1 M NaOH solutions: (**a**) specimen 1, (**b**) specimen 2, (**c**) specimen 3, and (**d**) specimen 4.

**Figure 9:**
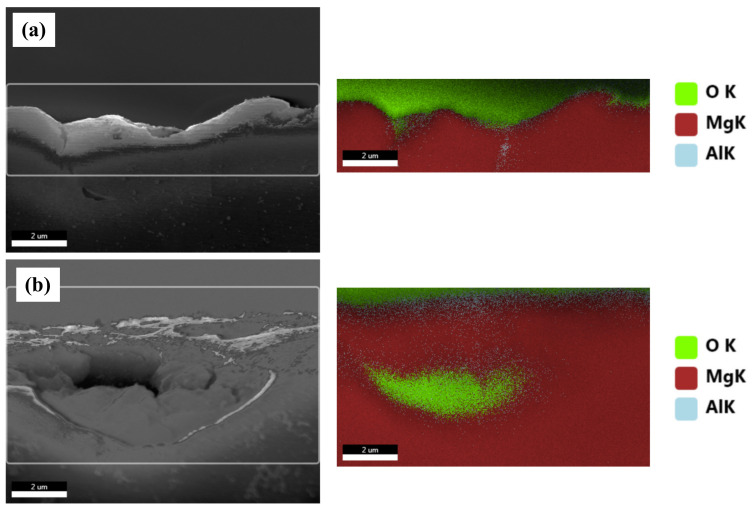
EDX analysis of the selected cross-sectional SEM images of the corroded specimens in 0.1 M NaOH solutions: (**a**) a general corroded surface and (**b**) a surface with a removed eutectic phase.

**Figure 10:**
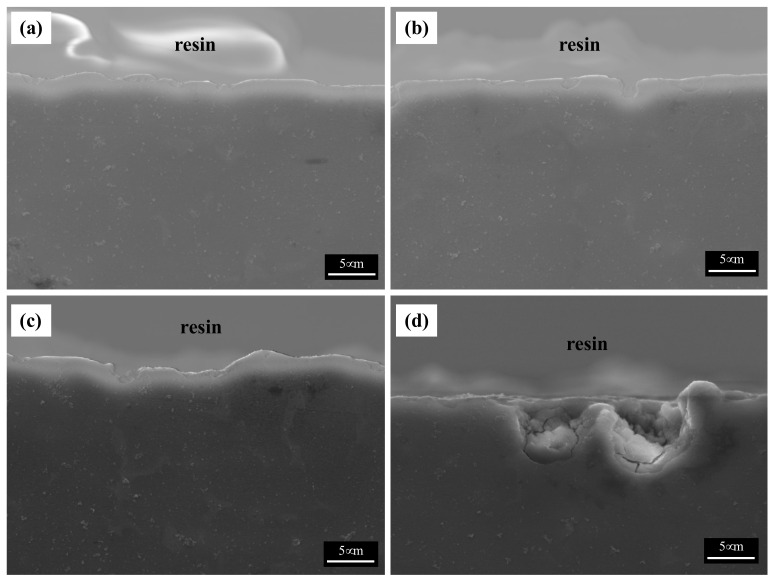
SEM images of the cross-section of the corroded specimens after corrosion tests in 0.1 M NaOH solutions: (**a**) specimen 1, (**b**) specimen 2, (**c**) specimen 3, and (**d**) specimen 4.

Figure 8 shows the BSE images of the corroded surfaces of the specimens in the alkaline solution following the corrosion tests. The magnified images of the corroded surfaces are also displayed in the insets of Figure 8a–d. The dark spots in the BSE images represent pits. Figure 8a shows the generation of small pits, which seem to be networked, as the corroded regions are composed of fine β phases that are densely distributed. Figure 8b shows larger pits (approximately 20 μm in diameter) than those in specimen 1, and crevice corrosion is also observed in the magnified corrosion regions. Figure 8c,d show deeply and largely corroded regions. Several pits were generated, as shown in Figure 8d. In the alkaline solution, most of the specimen surfaces were covered by a passive film, and some of the corroded regions were generated by galvanic corrosion, seen as pores in the images (pores are developed by the elimination of the β phase). During corrosion, the grains would dissolve preferentially, whereas most of the β-phase particles would be left on the surface, except for some that had been undermined and had fallen out because their surrounding areas had been preferentially dissolved, as the β-phase net acted as a barrier under chloride reaction. The net in hydroxide reaction also protects from galvanic corrosion and reduces the acceleration of the corrosion in the α matrix, as shown in Figure 8a. When the distance between the β phases increases, the β phases are positioned individually, and galvanic corrosion turns to dominant corrosion reaction so that the interface between the α matrix and β phase is corroded, leading to the β phase falling out. In hydroxide reaction, the net structure is more effective on corrosion resistance than chloride reaction. According to the corrosion results, the value of the corrosion current density of specimen 1 is extremely decreased, compared with specimen 4. Figure 9 shows the cross-sectional SEM images of the corroded surface of the specimens in the alkaline solution. As can be seen in the microstructure shown in Figure 9a, corrosion products and the eliminated eutectic phases on the surface of specimen 2 were difficult to observe. However, as the thickness increased, the regions with the eliminated phases gradually increased. The chemical composition of a typical microstructure was also analyzed by EDX mapping, and the results are shown in Figure 10. As the EDX results of the microstructure shown in Figure 10a indicate, 20 wt.% oxygen and 11 wt.% aluminum were detected on the surface. The region from which the phase was removed, shown in Figure 10b, was composed of 14 wt.% oxygen and 9 wt.% aluminum.

## 4. Conclusions

This study investigated the effects of cooling rates and the resulting microstructures of die-cast AZ91 specimens on mechanical properties and corrosion behaviors in both neutral and alkaline solutions. The following observations were made:

(1) The solidification rate was found to increase with decreasing specimen thickness, leading to the formation of finer β phases with shorter interphase distances, thus improving hardness. Additionally, these size distributions of the β phases were also found to affect the corrosion behavior.
(2) In a neutral solution, the coarse β phases in thick specimens led to an acceleration of the galvanic corrosion, producing large corrosion products.
(3) In a neutral solution, the fine phases in thin specimens interconnected to form a net structure act as an anodic barrier thereby reducing the corrosion rate. Specimens with fine phases showed widely and uniformly corroded regions, whereas specimens with coarse phases showed many large pits leading to crevice corrosion.
(4) In an alkaline solution, the corrosion behavior of all specimens indicated that the passivation films formed on the surface block attacking chloride ions, thus impeding electron migration between the phase and the matrix.
(5) The thinner specimens with finer β phases exhibited superior corrosion properties (with a higher corrosion potential than that of an Mg matrix), as galvanic corrosion hardly occurs in an alkaline solution.

According to the corrosion tests, the corrosion regions of the specimens were shown to be formed by phase distribution. Thus, the specimens with coarse phases showed large pits with sizes corresponding to the size of the phase.

## Figures and Tables

**Table 1:** Thickness, cooling rate, and the average distance between β phases of die-cast specimens.

Specimen	Thickness (mm)	Cooling Rate (°C/s)	Inter-Phase Distance (μm)
1	2.5	9.4	4.4
2	4	4.8	9.1
3	6	3.2	11.9
4	10	1.5	20.1

**Table 2:** Electrochemical parameters of die-cast specimens.

Specimen	Open-Circuit Potential (V)		E_0_ (V)		I_0_ (mA/cm2)	
	0.1 M NaCl	0.1 M NaOH	0.1 M NaCl	0.1 M NaOH	0.1 M NaCl	0.1 M NaOH
1	−1.74	−0.45	−1.57	−0.86	1.2	0.000025
2	−1.76	−0.55	−1.59	−1.06	1.5	0.0076
3	−1.77	−0.78	−1.62	−1.16	4.0	0.018
4	−1.78	−1.00	−1.64	−1.18	4.5	0.3

**Table 3:** Penetration rate (mm/py) of die-cast specimens.

	Specimen 1	Specimen 2	Specimen 3	Specimen 4
0.1 M NaCl	1.02	1.27	3.39	3.81
0.1 M NaOH	5.3 × 10^−7^	1.6 × 10^−4^	3.9 × 10^−4^	6.4 × 10^−3^

## Data Availability

Data are available in a publicly accessible repository.
